# Bioconversion of ferulic acid attained from pineapple peels and pineapple crown leaves into vanillic acid and vanillin by *Aspergillus niger* I-1472

**DOI:** 10.1186/s13065-020-0663-y

**Published:** 2020-02-03

**Authors:** Pei Ling Tang, Osman Hassan

**Affiliations:** 1 Department of Bioscience, Faculty of Applied Sciences, Tunku Abdul Rahman University College, Jalan Genting Kelang, Setapak, 53300 Kuala Lumpur, Malaysia; 2 School of Chemical Sciences and Food Technology, Faculty of Science and Technology, Universiti Kebangsaan Malaysia, UKM, 43600 Bangi, Selangor Malaysia

**Keywords:** Pineapple peel, Pineapple crown leaves, Vanillic acid, Vanillin, Ferulic acid, Bioconversion

## Abstract

This study was conducted to evaluate the potential of pineapple peel (PP) and pineapple crown leaves (PCL) as the substrate for vanillic acid and vanillin production. About 202 ± 18 mg L^−1^ and 120 ± 11 mg L^−1^ of ferulic acid was produced from the PP and PCL respectively. By applied response surface methodology, the ferulic acid yield was increased to 1055 ± 160 mg L^−1^ by treating 19.3% of PP for 76 min, and 328 ± 23 mg L^−1^ by treating 9.9% of PCL for 36 min in aqueous sodium hydroxide solution at 120 °C. The results revealed that PP extract was better than PCL extract for vanillic acid and vanillin production. Furthermore, the experiment also proved that large volume feeding was more efficient than small volume feeding for high vanillic acid and vanillin yield. Through large volume feeding, about 7 ± 2 mg L^−1^ of vanillic acid and 5 ± 1 mg L^−1^ of vanillin was successfully produced from PP extract via *Aspergillus niger* fermentation.

## Introduction

Pineapple is one of the main commodities in Malaysia. Its processing industry has been recognized as a highly potential industry by Ministry of Agriculture and Agro-based Industry of Malaysia (MOA). Pineapple industry is expected to expand at 5% per annum following the consistent acceleration of pineapple production. The export value of pineapple industry was predicted to increase from RM 161 mil. in year 2015 to RM 258 mil. by the year of 2020 [1]. However, the development of the pineapple processing industry has simultaneously contributed to the accumulation of agricultural residues such as pineapple peel, crown leaves, core etc. In pineapple canneries, nearly 75% of the fruits are discharged as waste [2]. The pineapple residues are commonly disposed by burning or decomposed due to the outdated technology and ignorance of the farmers and local entrepreneurs regarding the existence of commercial uses of pineapple residues [3]. Pineapple residues can be classified as on-farm waste such as leaves, stem, root remnants and processing waste such as core, peels, pulp etc. In overall, about 15.7–20.5 tonnes of total residues (dry basis) are generated following 12–18 tonnes pineapples processing [4]. Therefore, recycling of these potential lignocellulosic agricultural residues for the production of other value-added products is utterly needed to generate more revenues and overcome the environmental issues aroused from the improper by-products management in the industry.

Biotransformation of lignocellulosic by-products into vanillin by various fungi have been reported in several studies. Vanillin was reported being successfully produced from rice bran oil, corn cob, wheat bran, sugar beet pulp etc. by either native fungus such as *Aspergillus niger*, *Pycnoporus cinnabarinus*, *Pseudomonas fluorescens* etc. or bacteria such as *Escherichia coli* JM109/pBB1, *Bacillus aryabhattai* BA03 [5–10]. According to Kaur and Chakraborty [11], vanillin can be synthesized from a wide range of compounds such as eugenol, glucose, tyrosine, capsaicin, isoeugenol etc. by applying different fungi, bacteria, plant cell or genetically engineered microorganism etc. Among the proposed compounds, lignin, eugenol, isoeugenol and ferulic acid are the most potential substrates.

Ferulic acid is the most abundant hydroxycinnamic acid in plant. In herbaceous plant, it is bonded to lignin and hemicellulose via ester and ether links. Due to its abundancy, research on the biotransformation of this compound into high value vanillin is still on-going [6, 12, 13]. Ferulic acid extraction procedures was evolved with the objective to maximize the recovery of ferulic acid yield from the complex structure of lignocellulose for the high yield production of vanillin and other value-added compounds [14–17]. In this study, pineapple peel (PP) and crown leaves (PCL) were selected due to its abundancy in the local agricultural industry and its high ferulic acid content [18].

Most native microorganism tends to accumulate/excrete intermediate metabolites instead of vanillin during ferulic acid metabolism owing to the toxicity of vanillin towards cell metabolism. Nonetheless, basidiomycete such as *P. cinnabarinus* is more tolerant to vanillin [19]. However, ferulic acid polymerization reaction catalysed by the laccase enzyme secreted by *P. cinnabarinus* produces insoluble polymers during fermentation. In order to improve the vanillin yield, a two-pot bioconversion process of ferulic acid involving *A. niger* and *P. cinnabarinus* was initially proposed by Lasege-Meessen et al. [20]. However, the previous research was conducted by using ferulic acid and vanillic acid standard solution. Therefore, it is of great interest to determine the potential of ferulic acid-containing pineapple residues extracts in the production of vanillic acid and vanillin by *A. niger* alone in this study.

In this study, ferulic acid of pineapple peel (PP) and pineapple crown leaves (PCL) was initially extracted by using aqueous NaOH solution. The yield and recovery of ferulic acid and non-ferulate phenolic compounds (NFPC) from both PP and PCL were optimized through centre composite response surface methodology (CCDRSM). The vanillic acid and vanillin production by *A. niger* I-1472 were studied at different feeding modes. Eventually, the best substrate was selected based on the yield of vanillic acid and vanillin.

## Materials and methods

### Raw materials and fungal strain

PP and PCL of Morris pineapple used were purchased from the local hypermarket in Kajang, Selangor, Malaysia. The PP and PCL were prepared from the same batch of pineapple with the species labelling. The fresh PP was oven-dried at 105 °C for 12–14 h until the final moisture content reached approximately 5–8%, whereas the fresh PCL was oven dried (105 °C) for 6–8 h until its moisture content reached at 8–11%. Compositional analysis of PP and PCL was conducted according to the National Renewable Energy Laboratory (NREL) standard protocol [21–24]. The composition of PP and PCL were showed in Additional file 1: Appendix S1. The *A. niger I*-*1472* strain was purchased from the Collection Nationale de Culture de Microorganisms (CNCM), Institut Pasteus, Paris, France. The strain was maintained on malt extract agar (contained 20 g L^−1^ malt extract and 15 g L^−1^ agar) at − 40 °C. All other chemicals were analytical grade. Table 1.

**Table 1:** Centre composite response surface experimental design [actual and coded value (in bracket)] of the optimization of ferulic acid and NFPC from the black liquor of (a) pineapple peel (PP) and (b) pineapple crown leaves (PCL)

(a) Pineapple peel (PP)^a^					
Solid content (%)	Time (min)	Ferulic acid yield (mg L^−1^)	Ferulic acid recovery (mg g^−1^ PP)	NFPC yield (mg GAE mL^−1^)	NFPC recovery (mg GAE g^−1^ PP)
12.00 (0.000)	105.00 (0.000)	486.80	4.06	3.91	32.59
12.00 (0.000)	180.00 (1.414)	578.06	4.82	4.20	34.97
6.34 (− 1.000)	158.03 (1.000)	312.80	4.93	2.79	43.93
17.66 (1.000)	158.03 (1.000)	401.05	2.27	4.15	23.51
12.00 (0.000)	105.00 (0.000)	535.36	4.46	3.48	28.96
4.00 (− 1.414)	105.00 (0.000)	189.96	4.75	1.75	43.73
20.00 (1.414)	105.00 (0.000)	639.08	3.20	4.06	20.31
12.00 (0.000)	105.00 (0.000)	483.68	4.03	3.57	29.72
12.00 (0.000)	105.00 (0.000)	546.18	4.55	3.60	29.97
17.66 (1.000)	51.97 (− 1.000)	634.65	3.60	4.50	25.46
6.34 (− 1.000)	51.97 (− 1.000)	36.99	0.58	2.28	35.88
12.00 (0.000)	30.00 (− 1.414)	190.52	1.59	3.60	29.97
12.00 (0.000)	105.00 (0.000)	527.93	4.40	3.25	27.09

(b) Pineapple crown leaves (PCL)^b^					
Solid content (%)	Time (min)	Ferulic acid yield (mg L^−1^)	Ferulic acid recovery (mg g^−1^ PCL)	NFPC yield (mg GAE mL^−1^)	NFPC recovery (mg GAE g^−1^ PCL)
6.00 (0.000)	105.00 (0.000)	184.55	3.08	2.96	49.30
6.00 (0.000)	180.00 (1.414)	165.28	2.75	2.83	47.24
6.00 (0.000)	105.00 (0.000)	175.00	2.92	3.28	54.69
8.83 (1.000)	158.03 (1.000)	249.89	2.83	3.79	42.87
2.00 (− 1.414)	105.00 (0.000)	9.48	0.47	1.15	57.57
6.00 (0.000)	105.00 (0.000)	173.36	2.89	3.08	51.36
6.00 (0.000)	105.00 (0.000)	160.54	2.68	2.91	48.51
6.00 (0.000)	105.00 (0.000)	165.00	2.75	2.98	49.62
6.00 (0.000)	30.00 (− 1.414)	201.26	3.35	2.86	47.72
8.83 (1.000)	51.97 (− 1.000)	263.85	2.99	3.79	42.87
3.17 (− 1.000)	51.97 (− 1.000)	28.10	0.89	1.61	50.72
3.17 (− 1.000)	158.03 (1.000)	141.40	4.46	1.78	56.12
10.00 (1.414)	105.00 (0.000)	311.67	3.12	3.73	37.28

^a^mg GAE mL^−1^ and mg GAE g^−1^ PP means milligram of gallic acid equivalent per milliliter of extract and milligram of gallic acid equivalent per gram of pineapple peel respectively

^b^mg GAE mL^−1^ and mg GAE g^−1^ PP means milligram of gallic acid equivalent per milliliter of extract and milligram of gallic acid equivalent per gram of pineapple crown leaves respectively

### Aqueous alkaline treatment of PP and PCL

Black liquor of PP and PCL was obtained by treating 4% of PP and PCL respectively in 50 g L^−1^ aq. NaOH solution at 120 °C for an hour. Experiments were conducted in 500 mL Erlenmeyer flask in an autoclave. After the treatment, black liquor was separated from the residues by using muslin cloth under vacuum suction. All experiments were carried out in triplicate. The pH of the collected black liquor was adjusted to 2 by using concentrated sulphuric acid, followed by centrifugation at speed 1468×*g* for 5 min to recover the soluble lignin from the black liquor. Then, pH of the supernatant was adjusted to 5–6 by using calcium carbonate. Ferulic acid content in PP and PCL liquors was determined by using high performance liquid chromatography (HPLC), while total phenolic compounds content was determined through a modified Folin–Ciocalteau method [25].

Ferulic acid, vanillic acid and vanillin content were quantified using HPLC equipped with UV detector at wavelength 280 nm (Waters, Milford, USA). A 250 mm × 4.6 mm HPLC column Gemini C18 (Phenomenex, California, USA) with 5 μm particle size was used, and eluted using 0.1% acetic acid (solvent A) and methanol (solvent B) (Merck, Darmstadt, Germany). The mobile phase gradient was fixed at the ratio of 80:20 (A: B) from 0 to 24 min, 60:40 (A: B) from 24 to 27 min, 20:80 (A: B) from 27 to 36 min, and 80:20 (A: B) from 36 to 40 min. Flow rate of the analysis was fixed at 1 mL min^−1^. Mobile phase was filtered with 47 mm nylon membrane with pore size 0.45 μm and degassed. All the standards and samples were filtered through 0.22 μm nylon membrane prior to analysis. Vanillic acid, vanillin and ferulic acid purchased from Sigma Aldrich, Missouri, USA were used as the reference standards. Quantitative analysis was carried out by using caffeine (Sigma Aldrich, Missouri, USA) as an internal standard. Analysis was conducted with an injection volume of 10 μL and data acquisition was carried out with Breeze software (Waters, Milford, USA).

For total phenolic content (TPC) analysis, about 0.5 mL of 0.2 N Folin–Ciocalteau reagent (Merck, Darmstdt, Germany) was added into 0.1 mL aliquot. After 5 min conditioning at room temperature, about 0.4 mL of 75 g L^−1^ sodium carbonate solution (Merck, Darmstdt, Germany) was added, and the volume of the mixture was made up to 5 mL with distilled water. The mixture was then let to condition at room temperature in dark for 2 h. Absorbance of the mixture was determined spectrophotometrically at 760 nm using VERSAmax ELISA microplate reader coupled with SOFTmax Pro software (Molecular Devices, California, USA). TPC of the black liquor was quantified based on the constructed calibration curve using gallic acid (Sigma Aldrich, Missouri, USA) as the reference. Concentration of TPC was expressed as mg gallic acid equivalent per mL of liquor (mg GAE mL^−1^).

To determine the non-ferulate phenolic compounds (NFPC), the amount of ferulic acid (in mg GAE mL^−1^) was subtracted from the amount of TPC in the liquor. A series concentration of ferulic acid standard solutions and the samples were quantified by using HPLC (in mg L^−1^) and VERSAmax ELISA microplate reader (in mg GAE mL^−1^) respectively. The unit conversion curve of ferulic acid standard in unit mg mL^−1^ versus ferulic acid in unit mg GAE mL^−1^ was constructed. This conversion curve was then used to calculate the ferulic acid concentration of the samples in unit mg GAE mL^−1^.

### Optimization of ferulic acid and non-ferulate phenolic compounds (NFPC) production from PP and PCL

Ferulic acid yield from PP and PCL was optimized through central composite design (CCD) with 5 levels and 2 variables using Design Expert 6.0.10 software (Stat-Ease Inc., Minneapolis, USA). The effects of substrate concentration (X_1_) and treatment time (X_2_) on ferulic acid yield (Y_1_), ferulic acid recovery (Y_2_), NFPC yield (Y_3_) and NFPC recovery (Y_4_) were evaluated. The PP and PCL were treated in 50 g L^−1^ aq. NaOH solution at different substrate concentrations and times in an autoclave at 120 °C. The experimental design of ferulic acid extraction from PP and PCL is shown in Table 1a, b respectively. The polynomial models were developed to describe the relationship between the experimental variables (X_1_ and X_2_) and responses (Y_1_, Y_2_, Y_3_ and Y_4_). The optimum condition for maximum yield was predicted by integrating the constructed polynomial models. The main and interaction effects of the variables on the responses were evaluated based on the significance level of the regression coefficient in the model.

Because of the experiment limitation in lignin removal for black liquor produced from the treatment at higher solid content, ethyl acetate had been used as the ferulic acid extraction solvent. The failure in complete lignin removal from the produced liquor prevented it from HPLC analysis. An equal volume of ethyl acetate was added to the neutralized liquor, and shake vigorously at room temperature to recover the ferulic acid from liquor. The separation of these two layers was carried out under centrifugation at speed 1468×*g* for 3 min. The upper layer (ethyl acetate layer) was collected, and dried under vacuum using rotatory evaporator. The phenolic compounds were observed as the yellowish undissolved layer at the bottom of flask. The 50% v/v ethanol solution was then added to dissolve these compounds, followed by HPLC analysis according to protocol described in part 2.2.

Ferulic acid extraction with ethyl acetate was optimized using the black liquor produced from the optimized treatment. The extraction was repeated for two to three times by using an equal volume of ethyl acetate with the black liquor.

### Fermentation of PP and PCL extracts

*Aspergillus niger I*-*1472* was grown on malt extract agar (contained 20 g L^−1^ malt extract and 15 g L^−1^ agar) at 30 °C. The spores were harvested after 1 week of growth. About 1 mL of spore suspension (at concentration of 10^6^–10^7^ cfu mL^−1^) was inoculated into 100 mL basal medium containing 20 g L^−1^ maltose, 1.8 g L^−1^ diammonium tartrate, 0.5 g L^−1^ yeast extract, 0.5 g L^−1^ magnesium sulphate, 0.2 g L^−1^ dipotassium hydrogen phosphate and 0.013 g L^−1^ calcium chloride. The pH of medium was initially adjusted to 5. *A. niger I*-*1472* was incubated at 30 °C for 3 days with agitation at 150 rpm [26, 27]. About 0.5 mL of the neutralized PP and PCL liquors were fed daily to *A. niger* from day 3 until day 6. The medium was filtered with Whatman no. 1 filter paper at the end of fermentation to separate the cell pellets. Vanillic acid, vanillin and ferulic acid residue in the medium after fermentation were quantified by using HPLC as the described protocol in part 2.2.

The effect of liquor feeding modes, namely large volume feeding (LVF) and small volume feeding (SVF) on the vanillic acid and vanillin yield was investigated. For LVF, about 10 mL of PP and PCL liquor respectively was fed on day 3 and day 4. Prior to feeding, about 20 mL of medium was removed. The fermentation was stopped on day 5 by separating the cell pellets from fermentation medium through filtration. For SVF, about 1.5 mL of PP and PCL liquor respectively was fed daily from day 3 to day 6. Prior to feeding, about 6 mL of medium was removed. The fermentation was stopped on day 7. The fermentation control was fed with 300 mg L^−1^ and 1000 mg L^−1^ ferulic acid standard solution. Vanillic acid, vanillin and ferulic acid contents were determined using HPLC.

All experiments were conducted in triplicates. The results are expressed as mean ± standard deviation. The significant differences between the means were determined through one-way ANOVA, followed by Duncan post hoc test at 95% confidence interval using SPSS software version 20 (IBM Corporation, USA).

## Results and discussion

### Ferulic acid extraction

Alkaline treatment of PP and PCL with 50 g L^−1^ NaOH at 120 °C for an hour successfully yields 202 ± 18 mg L^−1^ and 120 ± 11 mg L^−1^ ferulic acid respectively. PP was initially proved as the best source of ferulic acid, where 5 mg of ferulic acid was produced from each gram of PP (in dry basis), while only 3 mg of ferulic acid was produced from each gram of PCL (in dry basis). Beside ferulic acid, other phenolic compounds were also extracted into the black liquor during alkaline treatment. About 0.74 ± 0.02 mg GAE mL^−1^ (with the recovery of 18.4 ± 0.6 mg GAE g^−1^ PP) and 0.66 ± 0.09 mg GAE mL^−1^ (with a recovery of 17 ± 2 mg GAE g^−1^ PCL) of NFPC had been extracted from PP and PCL respectively.

As reported by Buranov and Mazza [12], hydroxycinnamic derivatives such as *p*-coumaric acid and ferulic acid are attached to the lignin and hemicellulose via ester and ether bonds as a bridge in the herbaceous plant. Evidence of the esterification of ferulic acid to hemicellulose of pineapple cell wall was also reported by Smith and Harris [28]. In order to release these valuable components from the lignin/phenolics–carbohydrate complexes of the pineapple residues, alkaline solvent was chosen in this study. During alkaline hydrolysis, the alkali cleaves the ester bond of ferulate bridge, liberating the ferulic acid and lignin from the hemicellulose. Among various alkaline available, sodium hydroxide had been selected because it was reported to be more selective in the released of phenolic compounds [29].

As reported in this study, ferulic acid extracted from PP was higher than PCL. This finding can be explained by the differences in hemicellulose and lignin contents of PP and PCL (shown in Additional file 1: Appendix S1). As ferulic acid is presence as ferulate bridge in the lignin–carbohydrate structure, we strongly believed that there is a relationship between the ferulic acid content and hemicellulose and lignin content of the pineapple residues. Both hemicellulose and lignin content of PP is higher than PCL. Thus, PP was expected to contain higher ferulic acid.

In addition, the initial finding from a fermentation study on the black liquor by *A. niger* suggested that the production of vanillic acid was mainly due to the conversion of ferulic acid. Ferulic acid was disappeared while other unknown NFPCs were detected after fermentation. About 1.05 ± 0.05 mg L^−1^ (at molar conversion of 9%) and 0.5 ± 0.1 mg L^−1^ (at molar conversion of 7%) of vanillic acid had produced from the fermentation of neutralized PP and PCL liquor respectively. Biotransformation of ferulic acid by *A. niger* I-1472 into vanillic acid, prior to vanillin production by *Pcynoporus cinnabarinus* MUCL 39533 is a well-known fermentation process by Lesage-Meessen et al. [20]. In their study, the productivity of vanillic acid was reported at molar conversion of 88% with the feeding of 300 mg L^−1^ ferulic acid. However, the productivity of vanillic acid from the black liquor of pineapple residues was not as efficient as those fed with the ferulic acid standard solution. The lower molar conversion rate of ferulic acid in the fermentation of pineapple residues black liquors was suspected due to some extent of inhibition by the unknown NFPC and other toxic compounds, as well as the lower concentration of ferulic acid in the liquors. Therefore, in order to optimize the productivity of vanillic acid, optimization of ferulic acid yield from the pineapple residues and the study on the effects of feeding modes on the efficiency of fermentation were carried out in the following study.

### Optimization of ferulic acid and NFPC extraction

The ferulic acid and NFPC yield and recovery from PP and PCL through different treatment conditions were showed in Table 1a, b. The influences of each variable (X_1_: substrate concentration; X_2_: treatment time) on the responses (Y_1_: ferulic acid yield; Y_2_: ferulic acid recovery; Y_3_: NFPC yield; Y_4_: NFPC recovery) were explained by the developed regression models (as shown in Additional file 2: Appendix S2).

Table 2a, b showed the results of regression analysis on the regression coefficient of the models. Based on the results, most of the regression coefficient that explained the relationship between variables (X_1_ and X_2_), and responses (Y_1_, Y_2_, Y_3_ and Y_4_) were significant (*p *< 0.05) for both substrate PP and PCL. Besides, the interaction of X_1_X_2_ also showed significant (*p *< 0.05) for both Y_1_ and Y_2_. In addition, the results also indicated that all regression coefficients of variable X_1_ were significant (*p *< 0.05) for the model that explained NFPC yield and recovery, but not for variable X_2_. Therefore, this finding confirmed that the substrate concentration (X_1_) exerted a stronger effect than treatment time (X_2_) in the extraction of NFPC. These findings were supported by a similar study reported by Torre et al. [29], where the amount of ferulic acid released from the corn cobs was reported to increase following the increase of solid: liquid ratio in the treatment. Moreover, the ferulic acid and NFPC yield were also found to increase to a larger extent under high substrate treatment, compared to the long-time treatment.

**Table 2:** Regression analysis on the regression coefficient of polynomial models that explaine the influences of substrate concentration (X_1_) and treatment time (X_2_) on the yield and recovery of ferulic acid and NFPC from (a) pineapple peel (PP) and (b) pineapple crown leaves (PCL)

(a) Pineapple peel (PP)								
Coefficient	Ferulic acid				NFPC			
	Yield		Recovery		Yield		Recovery	
	Coefficient^a^	*p*-value^b^	Coefficient^a^	*p*-value^b^	Coefficient^a^	*p*-value^b^	Coefficient^a^	*p*-value^b^
Linear								
β_1_	+ 184.17	0.01	+ 0.72	0.27	+ 0.86	< 0.00	− 7.99	< 0.00
β_2_	− 115.91	0.06	+ 0.37	0.55	+ 0.13	0.13	+ 1.65	0.02
Quadratic								
β_11_	− 64.00	0.01	− 0.35	0.13	− 0.32	< 0.00	+ 1.16	0.09
β_22_	− 79.11	< 0.00	− 0.73	0.01	+ 0.17	0.06	+ 1.39	0.05
Cubic								
β_111_	− 12.69	0.69	− 0.64	0.14	–	–	–	–
β_222_	+ 126.47	0.01	+ 0.38	0.34	–	–	–	–
Interaction								
β_12_	− 127.35	< 0.00	− 1.42	< 0.00	− 0.21	0.08	− 2.50	0.01

(b) Pineapple crown leaves (PCL)								
Coefficient	Ferulic acid				NFPC			
	Yield		Recovery		Yield		Recovery	
	Coefficient^a^	*p*-value^b^	Coefficient^a^	*p*-value^b^	Coefficient^a^	*p*-value^b^	Coefficient^a^	*p*-value^b^
Linear								
β_1_	+ 65.28	< 0.00	− 0.70	0.07	+ 0.98	< 0.00	− 6.23	< 0.00
β_2_	+ 62.39	< 0.00	+ 1.92	< 0.00	+ 0.02	0.76	+ 0.59	0.49
Quadratic								
β_11_	− 5.84	0.13	− 0.44	0.01	− 0.28	< 0.00	− 1.46	0.14
β_22_	+ 5.51	0.14	+ 0.19	0.13	− 0.07	0.23	− 1.44	0.14
Cubic								
β_111_	+ 20.78	0.02	+ 0.82	0.01	–	–	–	–
β_222_	− 37.55	< 0.00	− 1.07	< 0.00	–	–	–	–
Interaction								
β_12_	− 31.82	< 0.00	− 0.93	< 0.00	− 0.04	0.57	− 1.35	0.28

Note: ^a^Coefficient indicates the regression constant of the linear, quadratic, cubic and interaction terms in the polynomial equation that explain the relationship between substrate concentration (X_1_) and treatment time (X_2_) with the yield and recovery of ferulic acid (Y_1_, Y_2_) and NFPC (Y_3_, Y_4_)

^b^*p*-value lower than 0.05 indicate that the effect of the term on the yield and recovery of ferulic acid and NFPC are significant at 95% confidence interval. *p*-value lower than 0.01 indicate that the effect of the term on the yield and recovery of ferulic acid and NFPC are significant at 99% confidence interval

However, the recovery of ferulic acid from PP and NFPC recovery from both PP and PCL were reduced with the increase of substrate concentration. Compounds (ferulic acid and NFPC) saturation in the solvent during treatment at high solid: liquid ratio was strongly believed to be the main factor that caused the reduction of ferulic acid and NFPC recovery. Nonetheless, this condition was not observed for PCL. This finding suggested that the substrate and ferulic acid saturation phenomenon did not occur in the PCL treatment. The lower substrate concentration (2–10%) and ferulic acid availability in PCL were probably the factors that prevent substrate and ferulic acid saturation phenomenon during PCL treatment. In spite of that, Mussatto et al. [30] reported that treatment time exerted stronger effect in improving ferulic acid yield than other phenolic compounds such as coumaric acid. The efficiency of a treatment to release phenolic compounds from lignin/phenolic–carbohydrate complex is strongly affected by the types and properties of chemical bonds in the structure [12, 31]. Therefore, due to the involvement of several types of chemical debonding during NFPC release, the NFPC extraction efficiency was least affected by the treatment time. Compared to NFPC extraction, ferulic acid extraction probably involved a more specific cleavage of chemical linkages.

The validity of the obtained regression models was analysed statistically by ANOVA. The insignificant *lack of fit* (*p *> 0.05) and high R^2^-value (r^2^ > 0.90) of the regression models suggested that the trend of changes of ferulic acid and NFPC yield and recovery from PP and PCL through different treatment condition can be well-explained by these regression equations. Thus, an optimum treatment condition with the highest ferulic acid and NFPC yield and recovery were predicted by using these regression equations. The optimum conditions for maximum ferulic acid and NFPC yield and recovery from PP and PCL were successfully obtained by integrating the regression equations. Upon optimization of PP, about 728 mg L^−1^ of ferulic acid (with the recovery of 4 mg g^−1^ PP) and 5 mg GAE mL^−1^ of NFPC (with recovery of 23 mg GAE g^−1^ PP) were predicted by treating 19.3% of PP in 50 g L^−1^ NaOH aqueous solution at 120 °C for 76 min. These predictions were successfully verified within 95% confidence interval with the detection of 766 ± 37 mg L^−1^ of ferulic acid (with recovery of 4.0 ± 0.2 mg g^−1^ PP) and 4.9 ± 0.2 mg GAE mL^−1^ of NFPC (with recovery of 25 ± 1 mg GAE mL^−1^) in the validation experiment.

Meanwhile, upon the treatment of 9.9% of PCL in 50 g L^−1^ NaOH aqueous solution at 120 °C for 36 min, about 369 mg L^−1^ of ferulic acid (with the recovery of 5 mg g^−1^ PCL) and 4 mg GAE mL^−1^ of NFPC (with the recovery of 39 mg GAE g^−1^ PCL) was expected to be produced. This prediction was also successfully verified within 95% confidence interval where 328 ± 23 mg L^−1^ of ferulic acid (with recovery of 3.3 ± 0.2 mg g^−1^ PCL) and 3.1 ± 0.3 mg GAE mL^−1^ of NFPC (with recovery of 32 ± 3 mg GAE g^−1^ PCL) were produced under this optimum condition.

Furthermore, the results of ferulic acid extraction using ethyl acetate showed that an equal volume of ethyl acetate extraction must carried out twice on PP liquor for complete ferulic acid extraction. About 1055 ± 160 mg L^−1^ of ferulic acid (with the recovery of 5.5 ± 0.8 mg g^−1^ PP) was successfully extracted. For PCL liquor which contained lower ferulic acid, single extraction by using an equal volume of ethyl acetate with the PCL liquor was sufficient for complete ferulic acid extraction.

Our results of this study revealed that RSM optimization improved the yield and recovery of ferulic acid and NFPC from PP and PCL significantly (*p *< 0.05). For PP, the ferulic acid yield and recovery had been improved approximately 423% and 8% respectively; while NFPC yield and recovery had improved 555% and 37% respectively after optimum treatment. While, the ferulic acid yield and recovery from PCL was also improved by about 174% and 11% respectively; whereas the NFPC yield and recovery were improved by 373% and 92% respectively after the optimum treatment. Furthermore, the improvement of ferulic acid extraction in this study was proven more effective than the previous study by Noor-Hasyierah et al. [32]. In their study by using substrate paddy straw, ferulic acid improvement after RSM optimization only achieved about 37%. Because of the ferulic acid and NFPC content of the black liquor determine its potential as a feedstock for vanillic acid (as well as vanillin) production in fermentation, optimization process became a crucial part in this study.

### Fermentation of PP and PCL liquor

Table 3 showed the yield and molar conversion of vanillic acid and vanillin from the PP and PCL liquors fermentation at two different feeding modes. The findings suggested that the vanillic acid and vanillin yield were significantly (*p *< 0.05) affected by the interplay between feeding modes and ferulic acid content of the feeding liquor. LVF contributed to higher vanillic acid and vanillin yield, compared to SVF. Fermentation of PP liquor which containing higher ferulic acid content produced higher vanillin yield (5 ± 1 mg L^−1^) than PCL liquor via LVF. Yet, the vanillic acid yield produced from the fermentation of PP and PCL liquor had no significant difference (*p *> 0.05). Nevertheless, trace vanillin (0.8 ± 0.1 mg L^−1^) was detected in the fermentation of PCL liquor at SVF, but not for PP liquor. In the SVF fermentation of PP liquor, vanillic acid (4 ± 2 mg L^−1^) was the only fermentation product. Furthermore, ferulic acid was completely consumed by *A. niger* in the SVF fermentation, but not for LVF. A large amount of unconsumed ferulic acid has been detected in the medium after LVF fermentation.

**Table 3:** Yield and molar conversion of the vanillic acid and vanillin from the fermentation of PP and PCL liquor in two different feeding modes (n = 3)

Product			Small volume feeding		Large volume feeding	
			PP liquor	PCL liquor	PP liquor	PCL liquor
Ferulic acid	Feed (μmol)		33 ± 5	10.1 ± 0.7	109 ± 17	34 ± 2
	Consumed (μmol)		33	10.1	48	18.4
	Remained	mg L^−1^	n/d	n/d	117 ± 10	30 ± 1
		μmol	< 0.00	< 0.00	60 ± 5	15.4 ± 0.5
Vanillic acid	Yield^a^	mg L^−1^	4 ± 2	0.9 ± 0.1	7 ± 2	6 ± 1
		μmol	2.6 ± 0.9	0.52 ± 0.07	3.8 ± 0.9	3.8 ± 0.7
	Molar conversion (%)^b^		7.9	5.1	7.9	20.7
Vanillin	Yield^a^	mg L^−1^	n/d	0.8 ± 0.1	5 ± 1	2.5 ± 0.2
		μmol	n/d	0.51 ± 0.06	3 ± 0.8	1.6 ± 0.2
	Molar conversion (%)^b^		n/d	0.5	6.3	8.9

*n/d* not detected

^a^Yield indicates the concentration (mg L^−1^) of vanillic acid/vanillin produced from *A. niger* fermentation

^b^Molar conversion indicates the percentage of vanillic acid/vanillin molar yield produced from the molar consumption of ferulic acid during *A. niger* fermentation

Although the vanillin yield from LVF fermentation of PCL liquor (2.5 ± 0.2 mg L^−1^) was lower than those fed with PP liquor (5 ± 1 mg L^−1^), its ferulic acid molar conversion was comparatively high. In LVF fermentation, only 45% and 54% of the total ferulic acid in the PP and PCL liquor respectively had been consumed. The molar conversion of vanillic acid and vanillin achieved 20.7% and 8.9% respectively during PCL liquor fermentation, while the molar conversion of vanillic acid and vanillin from ferulic acid in PP liquor was only about 7.9% and 6.3% respectively. These results unveiled that PCL liquor had better fermentability than PP liquor. Nevertheless, PP liquor was a better substrate than PCL liquor for higher vanillin yield. Based on these findings, our initial hypothesis that propose the interplay between feeding modes and ferulic acid concentration on the yield and molar conversion of vanillic acid and vanillin in *A. niger* fermentation was supported. In addition, the molar conversion of vanillic acid from PP liquor in both SVF and LVF was insignificant (*p *> 0.05). However, the molar conversion of vanillic acid of PCL liquor was increased fourfold when the feeding mode was changed from SVF to LVF.

Table 4 showed the comparison of vanillic acid yield and its molar conversion in the fermentation fed with ferulic acid standard solution, PP and PCL liquors. The results obtained indicated that only fermentation fed with PP and PCL liquors produced vanillin. Nonetheless, the vanillic acid yield and molar conversion in the fermentation of PCL liquor was higher than with fermentation of 300 mg L^−1^ ferulic acid standard solution. On the other hand, the vanillic acid yield and molar conversion in the fermentation of PP liquor was lower than with fermentation of 1000 mg L^−1^ ferulic acid standard solution. This finding suggests that the NFPC content in the liquors of PP and PCL significantly affects the vanillic acid and vanillin production by *A. niger*. The interplay of the feeding modes, ferulic acid and NFPC concentration was strongly believed to be the factor that contributed to this observation, possibly by an effect on the metabolic pathway of *A. niger*.

**Table 4:** Vanillic acid yield and its molar conversion during the fermentation of ferulic acid standard solution and liquors of pineapple residues (PP and PCL) (n = 3)

Substrate	Yield (mg L^−1^)^a^	Molar conversion (%)^b^
Small volume feeding		
300 mg L^−1^ ferulic acid	< 0.00	0
PCL liquor	0.9 ± 0.1	5.1
1000 mg L^−1^ ferulic acid	5.8 ± 0.7	11.2
PP liquor	4 ± 2	7.9
Large volume feeding		
300 mg L^−1^ ferulic acid	3.9 ± 0.7	8.2
PCL liquor	6 ± 1	20.7
1000 mg L^−1^ ferulic acid	15 ± 2	11.2
PP liquor	7 ± 2	7.9

^a^Yield indicates the concentration (mg L^−1^) of vanillic acid/vanillin produced after *A. niger* fermentation

^b^Molar conversion indicates the percentage of vanillic acid/vanillin molar yield produced from the molar consumption of ferulic acid during *A. niger* fermentation

**Fig. 1:**
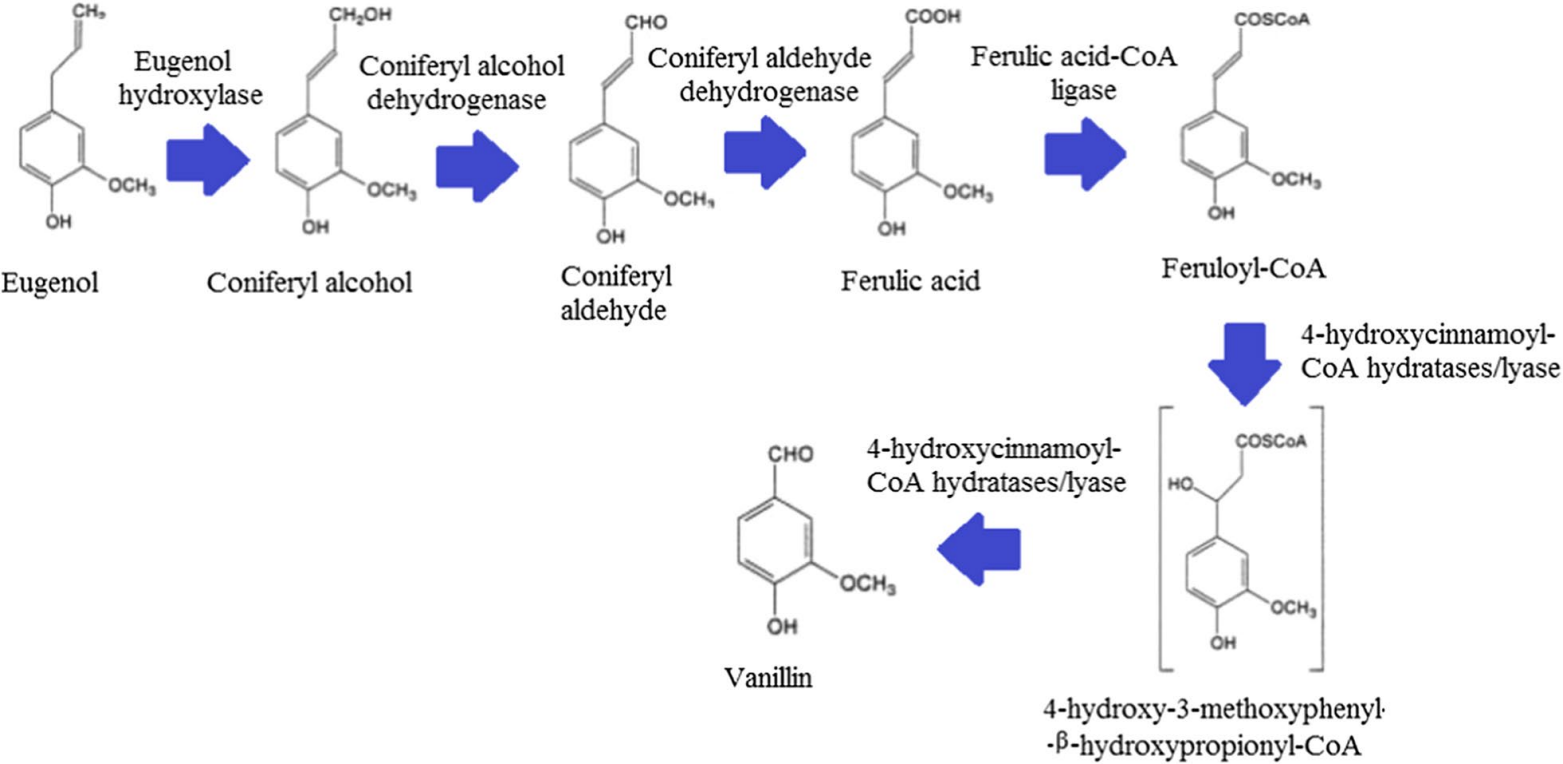
The proposed metabolic pathway of *Aspergillus niger* in vanillin production from eugenol by Srivastava et al. [33]

PP liquor contained higher ferulic acid and NFPC content than PCL liquor. Therefore, the impact of NFPC on the fermentability of PP liquor was likely stronger than PCL liquor. According to the study by Srivastava et al. [33], *A. niger* was expected to produce vanillin from eugenol through a metabolic pathway with ferulic acid as an intermediate metabolite (as shown in Fig. 1). This metabolic pathway was constructed based upon the reference pathway in *P. fluorescens*. During ferulic acid metabolism by *P. fluorescens*, vanillic acid is secreted as the end product following the action of vanillin dehydrogenase enzyme in the bioconversion of vanillin [10]. Therefore, assuming that *A. niger* followed a similar ferulic acid metabolic pathway as *P. fluorescens*, the vanillin dehydrogenase enzyme expression during ferulic acid metabolism was believed to be the factor that caused the accumulation of vanillic acid during the ferulic acid fermentation by *A. niger*. As reported by Lindahl [34], aldehyde compounds rarely accumulate as an end product in the biological system because of its high chemical reactivity. Therefore, various enzyme systems exist simultaneously in the cell metabolism to metabolize aldehyde into less reactive derivatives such as carboxylic acid.

During the PP and PCL liquors fermentation, the interplay of feeding modes, ferulic acid and NFPC content in the feeding liquors may affect the accessibility of ferulic acid and NFPC to *A. niger*. The responses of *A. niger* to its environmental changes may disturb the activation/inhibition mechanism of its enzyme system in the cell. PP and PL liquors which are rich in various phenolic compounds may exert some extra effects on the cell physiology and its metabolic pathway. Therefore, we strongly believed that the combined effects of high ferulic acid and NFPC content during LVF fermentation caused vanillin excretion as an overflow intermediate metabolite during ferulic acid metabolism. A similar finding was reported in the study by Muheim and Lerch [19], where *Streptomyces setonii* excreted vanillin as an overflow metabolite when the vanillic acid content reached 200 mg L^−1^. In our study, LVF of both PP and PCL liquor produced about 6.4 mg L^−1^ vanillic acid, but the yield of vanillin was different.

Direct formation of vanillin at high yield by means of biotechnological approaches was seldom reported, as vanillin is well-known to inhibit cell metabolism. Thus, most microbes possess a specific enzyme system that tends to oxidize or reduce the vanillin compound into derivatives such as vanillic acid, vanillyl alcohol, guaiacol, protocatechuic acid etc. [19, 35]. Although a definite ferulic acid metabolic pathway of *A. niger* still unclear, we strongly believed that the interplay of feeding modes, ferulic acid and NFPC content influenced the metabolic pathway of *A. niger* during its ferulic acid metabolism, leading to the production of vanillin. A high level of ferulic acid and NFPC loading during LVF fermentation probably stimulate the conversion of ferulic acid into vanillic acid, while facilitate the vanillin excretion as an overflow mechanism. This may explain why vanillin was only detected at a significant level in LVF fermentation for both PP and PCL liquors. According to Nicholas et al. [10], vanillin (or vanillic acid) can be produced through various biotechnological means by either single or mixed microorganisms. Various biosynthetic pathways and enzyme systems were reported to participate in this biotransformation process involving different microorganism. Therefore, further study on the genes and proteins involved in the metabolic pathway of *A. niger* during the ferulic acid bioconversion is necessary for a better understanding of the fermentation of this fungus.

In addition, the results also suggest that shifting of feeding mode from SVF to LVF did not improve the vanillic acid molar conversion, but stimulated the accumulation of vanillin in PP liquor fermentation. Nevertheless, the yield and molar conversion of both vanillic acid and vanillin had improved significantly following the shift of the feeding mode from SVF to LVF. This observation further verified the influence of ferulic acid and NFPC concentration on the fermentation efficiency. According to Diana et al. [7], the efficiency of vanillin bioconversion from ferulic acid in crude wheat bran hydrolysate was lower, compared to the ferulic acid liquor that was recovered from the crude hydrolysate. Other compounds such as aldehyde or phenolic compounds that are present in the crude hydrolysate were reported to influence the performance of the microorganisms in fermentation, thereby reducing the molar yield and production of the end products. However, our results reveal that direct feeding of the biomass liquor has its pros and cons. Although the vanillic acid yield and molar conversion were reduced when feeding crude extract, vanillin yield was simultaneously enhanced. Thus, further studies profiling of the phenolic compounds in the pineapple residue liquor should be conducted, so that the influence of a defined compound on the *A. niger* metabolic pathway can be identified.

## Conclusion

This study proposes a novel use of the phenolic extracts of pineapple peel and crown leaves for the production of vanillic acid and vanillin. Compared to PCL liquor, PP liquor that containing the highest level of ferulic acid and phenolic compounds was shown to be the best substrate for vanillic acid and vanillin production. The interplay of feeding mode, ferulic acid and NFPC content was suggested to be an important factor affecting the efficiency of ferulic acid bioconversion into vanillic acid and vanillin. Furthermore, this study also shows that large volume feeding with liquor with a high ferulic acid content was more effective in enhancing vanillin production.

## Supplementary information

**Additional file 1.** Data of total composition of pineapple peel and pineapple crown leaves has been provided.

**Additional file 2.** Regression models for the CCDRSM optimization of ferulic acid and NFPC from pineapple peel (PP) and pineapple crown leaves (PCL).

## Data Availability

The datasets used and/or analysed in the current study are available from the corresponding author on reasonable request.

## Abbreviations

PP: Pineapple peel
PCL: Pineapple crown leaves
NFPC: Non-ferulate phenolic compounds
NREL: National Renewable Energy Laboratory
CNCM: Collection Nationale de Culture de Microorganisms
NaOH: Sodium hydroxide
HPLC: High performance liquid chromatography
GAE: Gallic acid equivalent
LVF: Large volume feeding
SVF: Small volume feeding
ANOVA: Analysis of variance
RSM: Response surface methodology
CCD: Central composite design
