# Supplementary material for: Bioconversion of ferulic acid attained from pineapple peels and pineapple crown leaves into vanillic acid and vanillin by Aspergillus niger I-1472
Source: BMC Chem. 2020 Feb 3;14(1):7. doi: 10.1186/s13065-020-0663-y (PMC6998299; doi:10.1186/s13065-020-0663-y)
Supplement: Supplementary file 1 — Additional file 1. Data of total composition of pineapple peel and pineapple crown leaves has been provided. [file 13065_2020_663_MOESM1_ESM.doc]

Appendix S1

Composition of pineapple peel (PP) and pineapple crown leaves (PCL) (n=3).

| Composition  (% dry basis) | PP | PCL |
| --- | --- | --- |
| Moisture | 8.54±0.08 | 11.4±0.2 |
| Ash | 5.5±0.3 | 7.8±0.3 |
| Total extractive | 45±1 | 40±3 |
| Total carbohydrate: | | |
| Cellulose (as glucan) | 13.4±0.1 | 29.08±0.05 |
| Hemicellulose (as xylan) | 26±3 | 18±1 |
| Total lignin: | | |
| Acid soluble lignin | 3.2±0.6 | 2.5±0.1 |
| Klason lignin | 16.5±0.2 | 12.8±0.7 |
