# Supplementary material for: Bioconversion of ferulic acid attained from pineapple peels and pineapple crown leaves into vanillic acid and vanillin by Aspergillus niger I-1472
Source: BMC Chem. 2020 Feb 3;14(1):7. doi: 10.1186/s13065-020-0663-y (PMC6998299; doi:10.1186/s13065-020-0663-y)
Supplement: Supplementary file 2 — Additional file 2. Regression models for the CCDRSM optimization of ferulic acid and NFPC from pineapple peel (PP) and pineapple crown leaves (PCL). [file 13065_2020_663_MOESM2_ESM.docx]

Appendix S2

Regression models for the CCDRSM optimization of ferulic acid and NFPC from pineapple peel (PP) and pineapple crown leaves (PCL)

For PP black liquor:

Y_1_=515.99+184.17X_1_-115.91X_2_-64.00X_1_^2^-79.11X_2_^2^-127.35X_1_X_2_-12.69X_1_^3^+126.47X_2_^3^ (R^2^=0.979)

Y_2_= 4.30+0.72X_1_+0.37X_2_-0.35X_1_^2^-0.73X_2_^2^-1.42X_1_X_2_-0.64 X_1_^3^+0.38X_2_^3^ (R^2^=0.940)

Y_3_=3.56+0.86X_1_+0.13X_2_-0.32X_1_^2^+0.17X_2_^2^-0.21X_1_X_2_ (R^2^=0.960)

Y_4_= 29.66-7.99X_1_+1.65X_2_+1.16X_1_^2^+1.39X_2_^2^-2.50X_1_X_2_ (R^2^=0.972)

For PCL black liquor:

Y_1_= 171.69+65.28X_1_+62.39X_2_-5.84X_1_^2^+5.51X_2_^2^-31.82X_1_X_2_+20.78X_1_^3^-37.55X_2_^3^ (R^2^=0.996)

Y_2_= 2.86-0.70X_1_+1.92X_2_-0.44X_1_^2^+0.19X_2_^2^-0.93X_1_X_2_+0.82 X_1_^3^-1.07X_2_^3^ (R^2^=0.970)

Y_3_= 3.04+0.98X_1_+0.02X_2_-0.28X_1_^2^-0.07X_2_^2^-0.04X_1_X_2_ (R^2^=0.982)

Y_4_= 50.70-6.23X_1_+0.59X_2_-1.46X_1_^2^-1.44X_2_^2^-1.35X_1_X_2_ (R^2^=0.903)

where X_1_ and X_2_ are referred as the main effect while X_1_X_2_ is the interaction effect of the variables. X_1_ represents PP/PCL concentration, X_2_ represents treatment time, Y_1_ represents ferulic acid concentration, Y_2_ represents ferulic acid recovery, Y_3_ represents NFPC concentration and Y_4_ represents NFPC recovery.
